# 95. The role of biologics in inflammatory arthritis: an interesting case

**DOI:** 10.1093/rap/rky032.003

**Published:** 2018-09-20

**Authors:** Babitha Mekkayil, Fiona Clarke

**Affiliations:** Rheumatology, James Cook University Hospital, Ingleby Barwick, UNITED KINGDOM

**Introduction:** Biologic therapies play an active role in the management of all inflammatory arthritis. They are usually well tolerated but should be routinely monitored for the high risk of infection and other associated side effects. Our case shows the importance of careful assessment of these patients.

**Case description:** This is a 55 year old lady with a background gut-associated arthropathy diagnosed in 2000. She was thoroughly investigated for inflammatory bowel disease and was diagnosed spastic colon and had subtotal colectomy and end ileostomy in the 1980s with rectal stump. She has failed multiple DMARDs in the past including, penicillamine, sulphasalazine, gold, leflunomide and methotrexate. She was one of our earliest patients to commence on biologics, with infliximab in 2001. Later she lost efficacy in 2003 and then changed to etanrecpt which caused a septic arthritis. This was successfully treated and later over the years tried adalimumab, abatacept and secukinumab in November 2017. Socially she lives with her husband and runs a family business. As she was losing efficacy to abatacept in November 2017 she was commenced on secukinumab and in December she presented to clinic with temporomandibular joint pain and flare of inflammatory arthritis. As she never responded well to steroids, this was managed with strong opioids. Two weeks later, she was admitted with fever and pelvic pain, seen by surgeons and rectal stump washout was done, discharged on the following day. This did not settle her symptoms and in next few days she was readmitted with overactive stoma, generally unwell, vomiting, dehydration and one stone weight loss. She was pyrexial at 38 degrees and had a dry blotchy rash with CRP of 154. She was commenced on empirical antibiotics and had a CT scan of her abdomen and pelvis. This has shown a few enlarged lymph nodes of unclear significance and ruled out intra-abdominal collections. She improved on antibiotics and CRP dropped down to 54. Later we have reviewed her as a ward referral, she described terrible joint pains in knees, ankles, shoulders and elbows and on examination had multiple tender joints, with full range of movement in her hip with pain. The secukinumab was stopped due to the allergic looking rash and she was given a course of oral prednisolone with an early follow up in two weeks. Within one week she presented to admission unit with severe hip pain and a groin abscess was suspected. A repeat CT abdomen and pelvis done at that time has shown a new psoas abscess, which was not present in the previous CT done 12 days back. She improved with the radiological drainage of the abscess and a prolonged three month antibiotic course. After successful treatment of the infection she was commenced on baricitinib and is doing very well on it.

**Discussion:** This case is a good example of risks and benefits in a patient with inflammatory arthritis. Biologics are like double edged sword which can manage the symptoms very well but they need a careful monitoring. Biologics helps in improved quality of life, fewer admissions to the hospital and fewer side effects from corticosteroids. The main concerns are high costs, risks of infections and uncertainty regarding long term safety. Infections and septicaemia is the most common cause of mortality. It can cause respiratory tract infections, urinary tract infections, mycobacterial infections and opportunistic infections etc. Studies have shown that standard-dose and high-dose biological drugs are associated with an increase in serious infections in rheumatoid arthritis compared with traditional DMARDs, although low-dose biological drugs are not. Past history of serious infections, glucocorticoid dose, and older age were other important predictors of risk of serious infections in patients treated with biologics.

**Key Learning Points**: This case is a good example of risks and benefits in a patient with inflammatory arthritis. It can manage the symptoms very well but they need careful monitoring.

**Disclosure: B. Mekkayil:** None. **F. Clarke:** None.

